
JOURNAL INFORMATION
==============================
NLM Title Abbreviation: Wirtschaftsdienst
Journal ID: Wirtschaftsdienst
NLM Journal ID: 101086612

Wirtschaftsdienst (Hamburg, Germany : 1949)

ISSN: 0043-6275

ARTICLE INFORMATION
==============================
PMCID: PMC8383023
PMID: 34456385
DOI: 10.1007/s10273-021-2977-3
Article ID: 2977
Article version: 1
Subjects: Zeitgespräch

Pandemiebereitschaft, internationale Kooperation und Marktdesign

Ockenfels Axel ockenfels@uni-koeln.de

grid.6190.e 0000 0000 8580 3777 Department of Economics, Universität zu Köln, Universitätsstraße 22a, 50923 Köln, Deutschland
Electronic publication date: 2021 Aug 24
Print publication date: 2021
Volume: 101
Issue: 8
First page: 594
Last page: 596
Copyright: © Der/die Autor:in(nen) 2021
License: Open Access: Dieser Artikel wird unter der Creative Commons Namensnennung 4.0 International Lizenz veröffentlicht (https://creativecommons.org/licenses/by/4.0/deed.de).
Open Access wird durch die ZBW — Leibniz-Informationszentrum Wirtschaft gefördert.
License URL: https://creativecommons.org/licenses/by/4.0/

issue-copyright-statement: © The Author(s) 2021

==============================

## Die Impfstoffproduktion während einer Pandemie erfordert entschiedene staatliche Interventionen

Krisen sind teuer. Für COVID-19 werden die globalen monatlichen Pandemiekosten auf 1.000 Mrd. Euro geschätzt, die deutschen Belastungen auf monatlich mehrere Milliarden Euro. Deshalb sind aus gesellschaftlicher Sicht fast alle Maßnahmen wünschenswert, die geeignet sind, Impfstoffe schneller zu entwickeln und zu produzieren. Dasselbe gilt jedoch nicht aus unternehmerischer Sicht. Unternehmen, die noch nicht wissen, ob ihr Impfstoffkandidat auch tatsächlich zugelassen werden wird und wie sich die Pandemie und mit ihr die Virusvarianten und die Notwendigkeit von Folgeimpfungen entwickeln, zögern mit teuren und riskanten Investitionen so lange, bis sich die Unsicherheiten möglichst auflösen. Im Ergebnis wird zu wenig und zu langsam in die Produktion von Impfdosen investiert.

Prinzipiell könnten die Preise für Impfstoffe im Bedarfsfall so stark hochschnellen, dass Unternehmen in der gesamten Lieferkette motiviert wären, Vorkehrungen zu treffen, um im Krisenfall schnellstmöglich produzieren zu können. Doch in der Krise ist die Politik als marktmächtige Nachfragerin aus ökonomischen, politischen und sozialen Gründen bestrebt, geringe Preise durchzusetzen. Während also Politik und Öffentlichkeit vor allem besorgt sind, einen zu hohen Preis zu zahlen, besteht aus ökonomischer Sicht die Gefahr, dass die Preise zu niedrig bleiben: Die Schätzungen des Wertes von Impfdosen zu Beginn der Pandemie lagen um bis zu tausendmal höher als die gezahlten Preise.

Doch dank staatlicher Anreizprogramme wurden die Impfstoffe gegen SARS-COV-2 schneller entwickelt und produziert, als es selbst die größten Optimisten zu Beginn der Pandemie für möglich gehalten haben. Vor 2020 gab es kein Unternehmen, das einen Impfstoff gegen SARS-COV-2 produziert hat. Im Jahr 2021 dürften mehr als 10 Mrd. Impfdosen hergestellt werden. Es gibt zwar immer noch keine ausreichende Impfstoffproduktion, um insbesondere die ärmeren Länder zu versorgen, doch dass die Impfstoffproduktion überhaupt in Rekordzeit skaliert werden konnte, geht auf die entschiedene Politik insbesondere in den USA und in England zurück. Kluges Marktdesign spielte dabei eine zentrale Rolle. Eine wichtige Maßnahme war, Impfstoffe verschiedener Technologien in großen Mengen und zu hohen garantierten Preisen bereits zu einem Zeitpunkt einzukaufen, zu dem noch nicht absehbar war, welche Impfstoffe überhaupt funktionieren und eine Zulassung erhalten würden. Die resultierenden Anreize haben die Entwicklung und den Ausbau der Impfstoffproduktion sowie die Produktion der Vor- und Zwischenprodukte während der Pandemie massiv beschleunigt (Ahuja et al., 2021; Castillo et al., 2021).

## Marktdesign für eine langfristige, resiliente Impfstoffproduktion

Eine andere Frage ist, wie die Anreize für eine mittel- und langfristig resiliente Absicherung mit Impfstoffen ausgestaltet werden sollten. Die langfristige Resilienz oder Zuverlässigkeit eines Marktes ist für viele Ökonom:innen ein sonderbares Ziel. In Lehrbüchern der Wirtschaftswissenschaft spielt es keine Rolle, denn die Zuverlässigkeit der Versorgung wird üblicherweise durch den markträumenden Preis garantiert, zu dem alle Nachfragenden, die bei diesem Preis kaufen wollen, auch bedient werden können. Doch Krisenzeiten sind keine normalen Zeiten. In krisenanfälligen Sektoren wie Finanzmärkten und Strommärkten müssen besondere Spielregeln und Maßnahmen dafür sorgen, dass Marktmechanismen auch in höchster Not ihre Koordinationsfunktion übernehmen können. Das gilt auch für Märkte, bei denen der Preismechanismus in der Krise an seine ethischen, sozialen oder ökonomischen Grenzen stößt. Die angewandte Marktdesignforschung hat hierfür Werkzeuge entwickelt und in vielen Fällen erfolgreich erprobt (Cramton et al., 2020; Chen et al., 2021).

* Diese Arbeit ist in Teilen im Rahmen einer Zusammenarbeit mit der von der deutschen Bundesregierung eingesetzten „Task Force Impfstoffproduktion“ entstanden. Die hier geäußerten Einschätzungen und Vorschläge spiegeln meine Ansicht wider, nicht notwendigerweise die Ansicht der Task Force oder ihrer Mitglieder. Einige der hier vorgebrachten Argumente sind Ockenfels (2021) entnommen und werden dort vertieft. Ich bedanke mich beim European Research Council (ERC, im Rahmen des European Union Horizon 2020 research and innovation programme, GA No 741409 – EEC) und der Deutschen Forschungsgemeinschaft (DFG, im Rahmen der Exzellenzstrategie des Bundes und der Länder – EXC 2126/1– 390838866) für die Unterstützung meiner Forschung.

Für die Frage, wie Impfstoffproduktionskapazitäten langfristig gesteuert werden können, erweist sich besonders der Blick auf ähnlich gelagerte Herausforderungen in der Stromwirtschaft als hilfreich. Wie bei der Impfstoffproduktion reichen auch in modernen Strommärkten die Preise in normalen Zeiten nicht aus, um Investitionen in Produktionskapazitäten zu rechtfertigen, die in extremen Knappheitssituationen benötigt werden. Auch die in einer Krise resultierenden Knappheitspreise begründen keinen hinreichenden Anreiz. Knappheitspreise müssen reguliert werden und die regulierten Preise sind tendenziell zu gering – ein Phänomen, das in der Stromwirtschaft als *missing money* bezeichnet wird (Cramton et al., 2013).

In den vergangenen Jahrzehnten wurden deshalb verschiedene Marktdesigns entwickelt, die langfristig für genügend Reserven in Strommärkten sorgen sollen (Cramton und Ockenfels, 2012). Dabei gibt es zwei marktkonforme Konzepte. Bei dem Mengenansatz (Kapazitätsmarkt) setzt der Regulierer die benötigte Erzeugungskapazität fest und lässt dann in einem wettbewerblichen Auktionsverfahren die potenziellen Erzeuger Preisforderungen bieten. Bei dem Preisansatz (Energy-Only-Markt) setzt der Regulierer den Preis fest, der in einer extremen Knappheitssituation für Strom gezahlt wird, und lässt dann die potenziellen Stromproduzierenden entscheiden, wie viel Kapazitäten und Reserven sie errichten möchten. Im ersten Fall steuert der Staat die Reserven direkt, im zweiten Fall nur indirekt über den garantierten Knappheitspreis.

Analog existieren zwei marktbasierte Optionen für eine resiliente Impfstoffproduktion. Die erste Option ist die Ausschreibung von Kapazitäten, die zu Kapazitätszahlungen führt – eine Vorab-Kostenteilerstattung oder ein Investitionskostenzuschuss für Produktionsanlagen. Im Gegenzug verpflichten sich die Unternehmen, eine bestimmte Menge Impfdosen bei Abruf bereitzustellen. Durch den Mechanismus werden die Risiken des Aufbaus von Produktionsanlagen teilweise von den Unternehmen auf den Staat übertragen, *missing money* kompensiert und der Neubau, Ausbau sowie der Weiterbetrieb bestehender Anlagen koordiniert. Bei der zweiten Option wird ein hoher Leistungspreis für Impfdosen in einer gewissen Menge und Qualität im Bedarfsfall garantiert. Impfstoffhersteller sollen dadurch einen Anreiz erhalten, sich frühzeitig auf künftige Pandemien vorzubereiten.

Beide Optionen haben Vorteile, jedoch für sich genommen auch gravierende Nachteile (Ockenfels, 2021). Ein Nachteil von Kapazitätszahlungen ist, dass sie zwar die Bereitstellung von Produktionsanlagen für Impfdosen belohnen, aber nicht unbedingt die schnelle Bereitstellung von qualitativ hochwertigen Impfdosen. Dies ist besonders dann der Fall, wenn als Gegenleistung zur Kapazitätszahlung eine Lieferung der Impfdosen zu niedrigen Preisen oder gar zu Preisen unter Grenzkosten erwartet wird, sodass der Leistungsanreiz gering ist: Wer Produktionskapazität bezahlt, bekommt dafür Kapazität – jedoch noch nicht unbedingt den gewünschten Impfstoff. Kapazitätszahlungen könnten im Gegenteil besonders für die Unternehmen attraktiv sein, die nicht daran glauben, dass sie einen erfolgreichen und profitablen Impfstoff in den Markt bringen können. Analog standen auf manchen Strommärkten durch Kapazitätszahlungen zwar genügend Erzeugungsanlagen zur Verfügung, doch erwiesen sich manche Anlagen in der Krise als wertlos, weil etwa mangels Brennstoff kein Strom produziert werden konnte.

Hohe garantierte Leistungspreise können Anreize für die schnelle Lieferung von Impfdosen und der benötigten Vorprodukte setzen. Doch die Steuerung des Impfstoffangebots allein durch einen garantierten Leistungspreis ist eine fragile Angelegenheit: Der richtige Preis ist nicht bekannt und dürfte jedenfalls auf politischen Widerstand stoßen, denn Leistungspreise, die den gesellschaftlichen Wert der Vorsorge und Krisenversorgung abbilden, müssten sehr hoch sein. Außerdem zeigt die Erfahrung in Strommärkten, dass es Politik und Unternehmen schwerfällt, die Auswirkungen unsicherer und sehr selten auftretender Ereignisse rational zu antizipieren.

Ein hybrides Marktdesign balanciert die Vor- und Nachteile der beiden Optionen aus. Der Staat kauft im Rahmen eines wettbewerblichen Verfahrens eine durch physische Kapazität gesicherte Kaufoption für Impfstoffe. Die Produzenten erhalten dabei eine Kapazitätszahlung für die Vorhaltung möglichst modularer physischer Produktionskapazität, die für die Herstellung unterschiedlicher Impfstoffe geeignet ist und mit der günstigenfalls in nicht-pandemischen Zeiten andere Produkte hergestellt werden können. Im Gegenzug erhält der Staat eine Kaufoption über eine bestimmte Menge der (zuerst produzierten) Impfdosen, die nicht mehr als beispielsweise 40 % der gesamten bezuschussten Produktionskapazität ausmachen soll. Die Auslieferung der Impfdosen muss innerhalb eines Zeitfensters nach Aktivierung des Bedarfsfalls geschehen; die Aktivierung erfolgt typischerweise bereits vor Zulassung der Impfstoffe. Der bei Ausübung der Kaufoption zu zahlende Leistungspreis bemisst sich nach dem dann vorherrschenden Marktpreis der Impfdosen, um ausreichend Leistungsanreize und Wettbewerbsfähigkeit zu ermöglichen.

Ockenfels (2021) erläutert, wie das hybride Marktdesign im Detail aussieht, welche Vorteile es hat und welche Herausforderungen es überwinden muss. An dieser Stelle sei erwähnt, dass die Anreizmechanismen des hybriden Marktdesigns ständig auf die aktuellen Impfstofftechnologien und die biologischen Gefahrenlagen angepasst werden können und in Krisenzeiten nahtlos hochskaliert und danach wieder zurückgefahren werden können. Wichtig für die Effektivität des Marktdesigns ist, dass der tatsächliche Bedarf an Vorhaltekapazitäten für Impfstoffe sorgfältig ermittelt wird. Unter anderem sollten dafür analytische und datengetriebene Modelle über mögliche künftige Pandemielagen hinzugezogen werden und Prognosen über den Wert unterschiedlicher Portfolios von Impfstofftechnologien und Produktionsanlagen im internationalen Kontext erstellt werden (Snyder et al., 2020).

## Design internationaler Kooperation und Koordination

Internationale Kooperation ist essenziell für die nationale und globale Versorgung mit Impfstoffen. Sie ermöglicht den grenzübergreifenden Handel mit Vor- und Zwischenprodukten, erhöht die Nachfragemacht in den Verhandlungen mit Impfstoffproduzenten, beschleunigt die Impfstoffversorgung ärmerer Länder (aus humanitären Gründen und zur Eindämmung epidemiologischer Externalitäten), koordiniert die Diversifikation der Impfstofftechnologien und nutzt Skaleneffekte in der Produktion aus. Kooperation in der Pandemie ist auch im Interesse der Gesundheits-, Sicherheits- und Wirtschaftspolitik. Deshalb sollte eine nationale Pandemiestrategie mit den europäischen Partnern und darüber hinaus abgestimmt werden. Eine mögliche Plattform dafür ist COVAX (COVID-19 Vaccines Global Access) (Gemünden und Thiel, 2021).

Die zentrale Herausforderung für internationale Kooperation ist die Anreizkompatibilität – die Vermeidung von Impfstoffnationalismus (Usher, 2021; Cramton et al., 2017; Schmidt und Ockenfels, 2021). Solange sich die Kooperationsbemühungen auf die Verteilung von Impfdosen konzentrieren und diese extrem knapp sind, haben wir es nicht mit einem Kooperationsspiel zu tun, sondern mit einem tragischen Nullsummenspiel. Was der eine Staat hat, fehlt dem anderen. Geht es um Leben und Tod, ist die Bereitschaft der Länder zu teilen und zu kooperieren gering. So haben sich die reichen Länder große Mengen an Impfstoffen gesichert, sodass COVAX bisher nur enttäuschend geringe Mengen an ärmere Länder verteilen konnte (Wouters et al., 2021).

Wenn im Zeitablauf mehr und mehr Impfdosen bereitstehen, und sogar Überschussangebote verteilt werden können, gibt es zunehmend Potenzial für einen koordinierten Austausch, getrieben durch Skaleneffekte bei der Produktion sowie durch die Differenzierung der Impfstoffe. Hier kann eine zentrale Markt- und Matching-Plattform eine wichtige koordinierende Rolle bei der fairen Verteilung der Impfdosen einnehmen und das Horten von Impfstoffen eindämmen (Cramton et al., 2020 und die dort zitierte Literatur). Kooperation ist auch dann vielversprechend, wenn sie sich nicht nur der Verteilung knapper Impfstoffe, sondern auch der Ausweitung der Produktion widmet. Auch ein reiches Land kann nur auf vergleichsweise wenige Technologien setzen und deshalb von einer international koordinierten, geografischen Streuung der Risiken profitieren. Ärmere Länder mit eigener Impfstoffproduktion und Zugang zu relevanten Technologien ermöglichen eine resilientere und gerechtere globale Versorgung. Überhaupt hilft eine leistungsfähigere globale Impfstoffproduktion allen Ländern und besonders den ärmeren Ländern, die sich am Ende der Warteschlange befinden. Außerdem dämmt sie Impfstoffnationalismus (Handelsbarrieren, Horten von Impfstoffen etc.) wirksam ein. Das hybride Modell und die vorgestellten Anreizmechanismen sind nicht nur mit nationalen, sondern auch mit international koordinierten multilateralen Programmen zum Ausbau von Impfstoffproduktionskapazität vereinbar.

## Märkte in der Krise und für die Krise

In der Pandemie spielt Marktdesign eine zentrale Rolle bei der Beschleunigung der Impfstoffentwicklung und -produktion, bei der Ausgestaltung der Pandemiebereitschaft und bei den Herausforderungen, die sich bei nationalen und globalen Verteilungs- und Kooperationsproblemen ergeben. Zugleich gibt es noch wichtige Herausforderungen, etwa bei der Sicherstellung der Versorgung mit Vorprodukten, Therapeutika und Diagnostika sowie beim Design von Abkommen zum Ausgleich von nationalen und globalen Interessen.

Prof. Dr. Axel Ockenfels ist Professor an der Fakultät für Wirtschafts- und Sozialwissenschaften und am Center for Social and Economic Behavior (C-SEB) der Universität zu Köln.


Literatur

Ahuja, A., S. Athey, A. Baker et al. (2021), Preparing for a pandemic: Accelerating vaccine availability, NBER Working Paper, 28492.
Castillo J C Ahuja A Athey S Market design to accelerate COVID-19 vaccine supply Science 2021 371 6534 1107 1109 10.1126/science.abg0889 33632897
Chen, Y., P. Cramton, J. List und A. Ockenfels (2021), Market design, human behavior and management, Management Science, im Erscheinen.
Cramton, P., D. J. C. MacKay, A. Ockenfels und S. Stoft (Hrsg.) (2017), Global Carbon Pricing: The Path to Climate Cooperation, MIT Press.
Cramton P Ockenfels A Roth A E Wilson R B Borrow crisis tactics to get COVID-19 supplies to where they are needed Nature 2020 582 7812 334 336 10.1038/d41586-020-01750-6 32528113
Cramton P Ockenfels A Stoft S Capacity market fundamentals Economics of Energy and Environmental Policy 2013 2 2 27 46 10.5547/2160-5890.2.2.2
Cramton P Ockenfels A Economics and design of capacity markets for the power sector Zeitschrift für Energiewirtschaft 2012 36 2 113 134 10.1007/s12398-012-0084-2
Gemünden, M. und J. Thiel (2021), COVAX Needs a Political Future, Policy Perspectives, 9, 4.
Ockenfels, A. (2021), Marktdesign für eine resiliente Impfstoffproduktion, Perspektiven der Wirtschaftspolitik, 10.1515/pwp-2021-0031/html (13. Juli 2021).
Schmidt, K. M. und A. Ockenfels (2021), Focusing climate negotiations on a uniform common commitment can promote cooperation, Proceedings of the National Academy of Sciences, 118(11).10.1073/pnas.2013070118 PMC7980435 33836569
Snyder C M Hoyt K Gouglas D Designing pull funding for a COVID-19 vaccine Health Affairs 2020 39 9 1633 1642 10.1377/hlthaff.2020.00646 32701395
Usher A D A beautiful idea: how COVAX has fallen short The Lancet 2021 397 10292 2322 2325 10.1016/S0140-6736(21)01367-2 PMC8494620 34147145
Wouters O J Shadlen K C Salcher-Konrad M Challenges in ensuring global access to COVID-19 vaccines: production, affordability, allocation, and deployment The Lancet 2021 397 10278 1023 1034 10.1016/S0140-6736(21)00306-8 PMC7906643 33587887
